# Tooth transposition: a multidisciplinary approach

**DOI:** 10.1590/2177-6709.23.1.097-107.bbo

**Published:** 2018

**Authors:** Mirian Aiko Nakane Matsumoto, Maria Bernadete Sasso Stuani

**Affiliations:** 1 Universidade de São Paulo, Faculdade de Odontologia de Ribeirão Preto (Ribeirão Preto/SP, Brazil).

**Keywords:** Tooth transposition, Corrective Orthodontics, Tooth extraction

## Abstract

Tooth transposition is one of the most difficult dental anomalies to treat in the dental clinic. Several factors must be taken into account with a view of making the best decision. The aim of this study was to discuss treatment modalities for tooth transposition, their advantages and disadvantages. Additionally, it aims at presenting a clinical case of transposition between canine and lateral incisor in the upper quadrant on the right side. The treatment of choice was extraction of one transposed tooth. A multidisciplinary approach involving Orthodontics, Cosmetic Dentistry, and Periodontology was necessary to allow proper esthetic and functional outcomes to be achieved.

## RESUMO

A transposição é considerada uma das anomalias dentárias mais difíceis de se administrar clinicamente. Vários fatores devem ser considerados para que a melhor decisão seja tomada. Neste artigo, o objetivo foi discutir as modalidades de tratamento da transposição dentária, suas vantagens e desvantagens, e apresentar um caso clínico de transposição entre canino e incisivo lateral na hemiarcada superior direita. A opção terapêutica de escolha foi a extração de um dos dentes transpostos, tendo-se a necessidade de uma abordagem multidisciplinar com a participação da Ortodontia, Dentística e Periodontia, a fim de que adequados resultados estéticos e funcionais fossem alcançados.

## INTRODUCTION

Tooth transposition is a unique and severe condition of ectopic eruption. It is defined as an interchange in the position of two permanent adjacent teeth located at the same quadrant in the dental arch. Transposition can be complete, when the position of affected teeth is totally transposed; or incomplete, when only the crowns are transposed, while the roots remain in normal position.^1^

The etiology of tooth transposition has been reported as being the result of genetic influences in a multifactorial model involving mechanical interference, trauma, tooth buds in altered position, early tooth loss, and long-term retention of deciduous teeth.^2^ ^-^ ^6^

The incidence of transposition in the overall population is low (0.2% to 0.38%).^6^ ^,^ ^7^ ^,^ ^8^ It is most often found among women,^7^ with the majority of cases being in the maxilla (76%) of which 88% are unilateral.^9^ The canines are affected in 90% of transposition cases, most often relative to the first premolar (71%) or maxillary lateral incisor (20%).^7^

While it may be present both in the maxilla and mandible, transposition between the canine and maxillary first premolar (Mx.C.P1) is the most common, followed by lateral incisor transposed with maxillary canine (Mx.C.I2). There are no reports on transposition in deciduous dentition.^10^

In 1995, Peck and Peck^7^ categorized tooth transposition into five types, according to the affected teeth: first premolars transposed with canines, canines with lateral incisors, first molars with canines, lateral incisor with central incisor, and canine transposed with central incisor. 

No protocol has been established to treat tooth transposition. However, the literature reports the following options:^11^

1. Interceptive treatment: provided the condition is found at early stages, with patients being from six to eight years old, it is recommended that deciduous teeth be extracted. This procedure is aimed to guide transposed tooth eruption back to its normal position, while the space is kept with a lingual arch or a transpalatal arch. The approach is only possible with tipped teeth, with roots near the desired position. This is also known as pseudo transposition.
2. Alignment of teeth at transposed position: recontouring of incisal or occlusal surfaces is necessary, thus reshaping affected teeth.
3. Extraction of one or both transposed teeth followed by orthodontic correction: the procedure is recommended when transposed teeth present severe caries, have been subjected to trauma, present little periodontal support and insufficient space.^12^
4. Orthodontic correction of transposed teeth: it requires longer treatment time, increases the risk of root resorption and bone loss.^10^

In cases of canine transposed with premolar (Mx.C.P1) and canine with lateral incisor (Mx.C.I2), the treatment of choice has often been alignment of teeth in transposed position or extraction of one or both transposed teeth. Correcting tooth transposition is considered difficult and demands long treatment time.^13-16^ It is necessary to take not only root position and tipping into account, but also the amount of bone available at the site of tooth movement. Care should be taken regarding mechanics with a view of preventing damage to the buccal bone plate, occlusal interference and root resorption.

A few factors must be valued when choosing the best treatment modality:

1. Dental morphology: important for cases keeping transposition unchanged, since reshaping of teeth is necessary to ensure favorable esthetic outcomes.
2. Occlusion: malocclusion and the potential for proper group function or canine guidance exert some influence on treatment choice.^11^ Should replacing the canine with first premolar be the choice, maxillary first premolar roots must present with morphology that allows movement without causing fenestration resulting from the buccal root.
3. Facial aesthetics: facial profile is also paramount whenever extraction is taken as an alternative.
4. Stage of development and location of root apices: alveolar bone buccolingual width often times is not enough to provide support for two adjacent teeth moving in opposite direction, especially when both have completely erupted. This may cause iatrogenesis to the teeth, such as root resorption, and to periodontal tissues, such as gingival tissue cleft and recession.
5. Treatment time: treatment time necessary for correction or maintenance of transposition must be taken into account from the best cost-benefit standpoint.^11^

Ciarlantini and Melsen^17^ have described the treatment options for the complete transposition of canines transposed with premolars (M.C.P1) or with lateral incisors (Mx.C.12), particularly with appliances especially designed to provide the correct force system. The authors reported being advantageous to treat patients when the canine has not fully erupted. When transposed teeth have fully erupted and have been (nearly) completely aligned at the transposed position, keeping transposition unchanged would be recommended. Even if possible, correction is not always advisable from a cost-benefit standpoint.^17^

The transposition may be kept unchanged for a few reasons, namely: potential for root resorption and gingival recession, as well as difficulty in controlling orthodontic mechanics. The root resorption is oftentimes present before the treatment of the patients with impaction or transposition.^18^ Moreover, it is often more distinct at treatment completion, in which case the treatment time is usually longer.^19^

The aims of orthodontic treatment are to restore the correct occlusion and provide favorable facial aesthetics, thus maintaining joint and periodontal health, as well as health of tooth support structures. Nevertheless, in a number of cases, a multidisciplinary clinical approach is rendered necessary, so as to allow all aims to be achieved.^20^ ^-^ ^23^

The aim of this study is to discuss treatment modalities for tooth transposition, their advantages and disadvantages. Additionally, it aims at presenting a clinical case of transposition between canine and lateral incisor in the upper quadrant on the right side. 

## CASE REPORT

Female African American patient, 17 years old, sought orthodontic treatment with chief complaint of having her right maxillary canine tipped and apparent. The clinical examination revealed the patient in good general health and presenting a history of trauma caused to tooth #11 and loss of teeth #36 and #16. 

**Figure 1:**
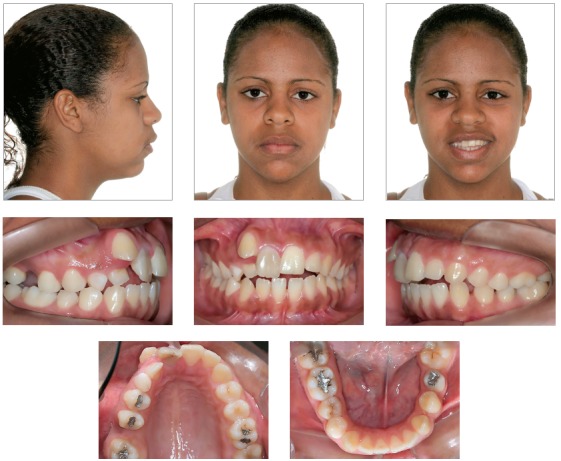
Initial facial and intraoral photographs.

In the frontal view, the patient presented a symmetrical face, increased lower third, dolichofacial pattern, and satisfactory lip seal with thick upper and lower lips. In the lateral view, she presented a convex profile, acute nasolabial angle, and obtuse cervico-mandibular angle. The smile line was inadequate due to the patient’s asymmetrical smile (Fig 1). 

The patient reported having the habit of biting objects. The functional pattern analysis evidenced mixed breathing, despite being predominantly oral, in addition to phonation and deglutition with anterior interposition of tongue. The tonsils, adenoids and TMJ were normal, with hyperactivity of both upper and lower lips. 

The intraoral examination revealed Class II canine relationship on the left side and impaired molar relationship due to loss of tooth #36. On the right side, the canine and molar relationship was also impaired due to tooth #12 being transposed with #13, as well as loss of tooth #16, respectively. Additionally, the patient presented anterior open bite, 3.5-mm overjet, maxillary anterior crowding, long-term retention of #53, darkened #11, loss of teeth #16 and #36, tooth #13 in labial-mesial infraversion, tooth #12 in palatoversion, and excess axial inclination of maxillary and mandibular incisors (bimaxillary protrusion) (Fig 1).

The dental cast assessments revealed - 2-mm discrepancy in the upper arch and null discrepancy in the lower arch, mild lower curve of Spee, 1-mm upper midline deviation to the right and 3-mm lower midline deviation to the left. Lower arch asymmetry was also found, with tooth #33 positioned 2.5mm distally to #43.

**Figure 2:**
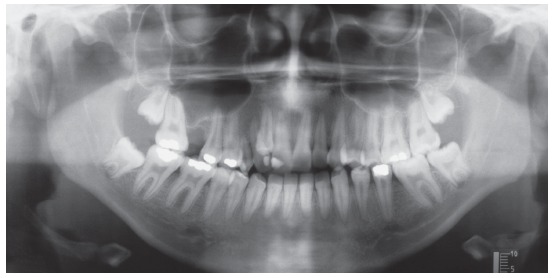
Initial panoramic radiograph.

**Figure 3:**
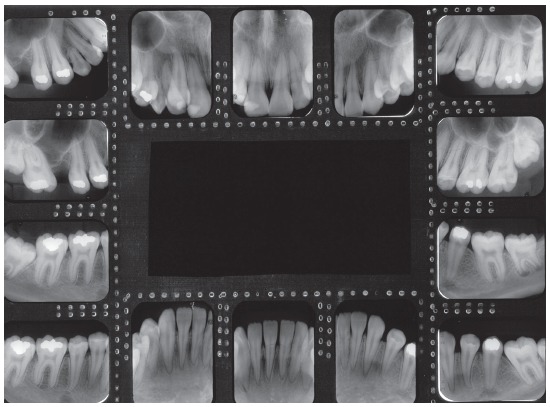
Initial periapical radiographs.

The panoramic radiograph confirmed absence of teeth #16 and #36, long-term retention of #53, and transposed #12 with #13. The trabeculae contour was normal, the lamina dura was intact for all teeth but #11, and bone loss with pneumatization of maxillary sinus was found in the region of tooth #16. Periapical radiograph also revealed root dilaceration of tooth #13 and radiolucent image in periapex of tooth #11 (Figs 2 and 3).

**Figure 4:**
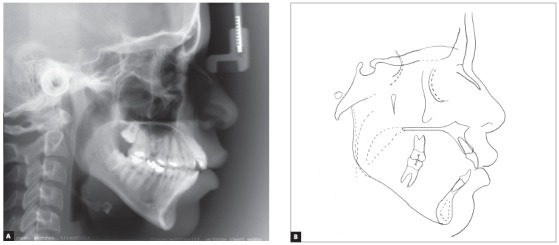
Initial lateral cephalogram (A) and cephalometric tracing (B).

The skeletal pattern was assessed by means of lateral cephalogram and revealed not only maxillary protrusion relative to the cranial base, but also a well-positioned mandible, thus evidencing a Class II skeletal pattern (SNA = 90°, SNB = 83° and ANB = 7°), with predominant facial vertical growth (SN.GoGn = 39° and Y-axis = 57^o^). Dental pattern revealed increased axial inclination and protrusion of both maxillary and mandibular incisors (1-NA = 8.5mm and 29°, 1-NB = 12mm and 38°). Lastly, a convex profile was found, with upper and lower lips positioned 7mm and 6mm forward relative to the S line, respectively (Fig 4, Table 1).

**Table 1:** Initial (A) final (B) cephalometric values.

	Measurements		Normal	A	B	Dif. A/B
Skeletal pattern	SNA	(Steiner)	82^o^	90^o^	85^o^	5
	SNB	(Steiner)	80^o^	83^o^	80^o^	3
	ANB	(Steiner)	2^o^	7^o^	5^o^	2
	Wits	(Jacobson)	♀ 0 ±2 mm ♂ 1 ±2 mm	8mm	1.5mm	6.5
	Angle of convexity	(Downs)	0^o^	13^o^	10^o^	3
	Y-axis	(Downs)	59^o^	57^o^	56.5^o^	0.5
	Facial angle	(Downs)	87^o^	93^o^	92^o^	1
	SN-GoGn	(Steiner)	32^o^	39^o^	40^o^	1
	FMA	(Tweed)	25^o^	27^o^	27^o^	0
Dental pattern	IMPA	(Tweed)	90^o^	99^o^	87^o^	12
	1.NA (degrees)	(Steiner)	22^o^	29^o^	18^o^	11
	1-NA (mm)	(Steiner)	4 mm	8.5mm	4.5mm	4
	1.NB (degrees)	(Steiner)	25^o^	38^o^	25^o^	13
	1-NB (mm)	(Steiner)	4 mm	12mm	8mm	4
	- Interincisal angle	(Downs)	130^o^	108^o^	133^o^	25
	1-APo	(Ricketts)	1 mm	8mm	4mm	4
Profile	Upper lip - S-line	(Steiner)	0 mm	7mm	4mm	3
	Lower lip - S-line	(Steiner)	0 mm	6mm	3.5mm	2.5

### Treatment plan and mechanics of choice

The orthodontic planning consisted of correcting transposition by means of extracting tooth #12, replacing the transposed tooth by the right maxillary canine (#13). Tooth #24 was extracted with a view of achieving Class I canine relationship and correcting protrusion of maxillary incisors. The extraction of #44 was recommended to correct lower midline deviation and decrease protrusion of mandibular incisors. As a result, a Class I molar relationship was achieved on the right side, while a Class II was kept unchanged on the left side. Retraction arches associated with intermaxillary elastics mechanics were used for the retraction of the maxillary and mandibular incisors. The ideal torque was introduced to the archwires used in coordination, so as to achieve functional occlusion. Furthermore, a multidisciplinary approach including endodontic treatment, tooth #11 bleaching, reshaping of tooth #13, and phonological therapy was planned.

## RESULTS

**Figure 5:**
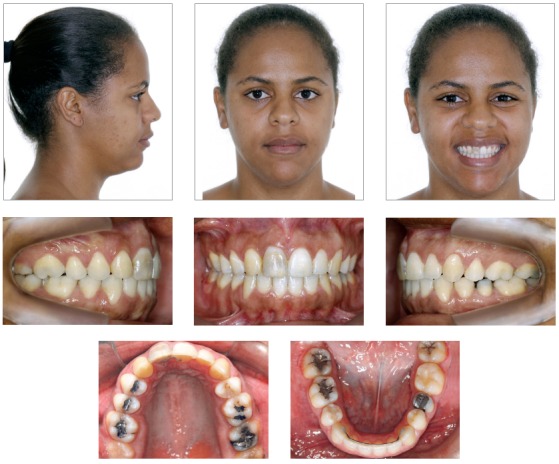
Final facial and intraoral photographs.

The treatment objectives were fulfilled, as shown by assessment of results achieved after the orthodontic treatment was carried out. The facial profile improved due to decreasing protrusion of the maxillary and mandibular incisors (Fig 5). 

In terms of tooth positioning, there were significant uprighting and retraction of maxillary and mandibular incisors, in addition to the correction of the anterior open bite. The lower arch asymmetry was improved and space between #17 and #15 closed, despite significant difficulty in uprighting tooth #17. 

Particularly in terms of function, respiratory pattern was improved as a result of the treatment carried out by the otolaryngologist. Moreover, the anterior interposition of the tongue while speaking and swallowing was corrected, as well as the lip tonicity as a result of myofunctional therapy. Proper occlusion was achieved from a functional perspective, with incisal guidance during protrusive movement of the mandible and disocclusion of canines on the left side (working occlusion), without balancing interference during lateral guidance. On the right side, group disocclusion was aimed due to tooth #14 replacing tooth #13. Therefore, functional occlusion and esthetic outcomes were achieved (Fig 5). 

**Figure 6:**
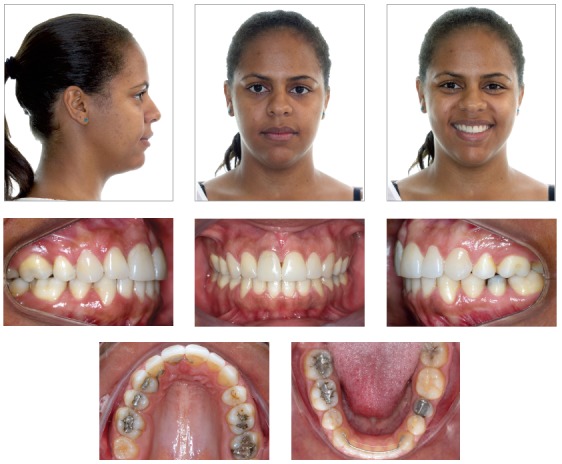
Facial and intraoral photographs taken after restorative treatment.

Subsequently, the patient was subjected to gingivoplasty to have gingival margins of teeth #13 and #14 restored. She also underwent restorative treatment of the maxillary incisors carried out by means of ceramic veneers, so as to have aesthetics improved (Fig 6).

**Figure 7:**
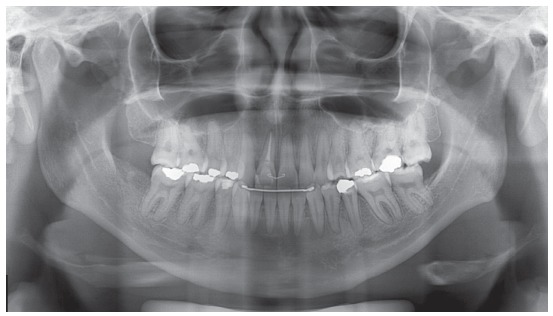
Final panoramic radiograph.

The panoramic radiograph revealed satisfactory parallelism between tooth roots and discreet apical remodeling of maxillary and mandibular teeth (Fig 7).

**Figure 8:**
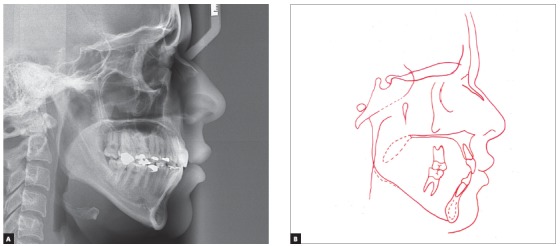
Final lateral cephalogram (A) and cephalometric tracing (B).

The skeletal pattern assessment revealed although the patient no longer presented facial growth, the ANB angle decreased in 2°. This probably occurred due to retraction of incisors and considerable decrease of axial inclination, especially relative to mandibular incisors (1.NB decreased in 13°, from 38° to 25°), which might have changed the position of point B (Fig 8, Table 1).

**Figure 9:**
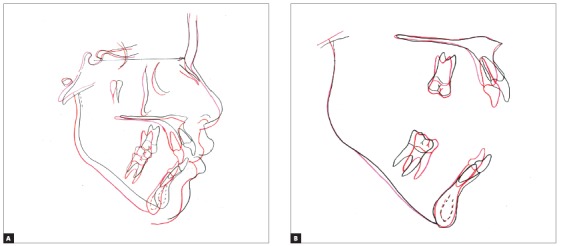
Total (A) and partial (B) superimposition of initial (black) and final (red) cephalometric tracings.

The total cephalometric superimposition revealed clockwise rotation of the mandible, opening of mandibular plane, decrease in protrusion of maxillary and mandibular incisors, and improved lip position. The partial maxillary superimposition revealed mild extrusion and decreased protrusion of maxillary incisors, with more significant palatal movement of the crown. The partial mandibular superimposition revealed mild extrusion and decreased protrusion of mandibular incisors, with more significant movement of the crown lingually. Molar was more significantly uprighted without extrusion (Fig 9).

## DISCUSSION

Tooth transposition is an anomaly difficult to be solved when the aim is to achieve success in orthodontic treatment. Thus, the orthodontist must reach accurate diagnosis and come up with a meticulous and comprehensive treatment plan. Most of the time, a multidisciplinary intervention is necessary. On the other hand, as the prevalence of the condition is relatively low, there is a lack of studies to aid determining not only the most effective treatment modality, but also the most convenient moment to begin.^24^

Several factors influence treatment choice: dental arch, affected teeth, location of crowns and roots, degree of resorption, malocclusion, clinician’s experience, and patient’s motivation.^11^ As for the patient reported herein, malocclusion hindered both aesthetics and function, which were extremely impaired due to the loss of permanent first molars (#16 and #36), presence of anterior open bite, protruded maxillary and mandibular incisors, as well as negative discrepancy in the maxilla. The transposition was complete.

In view of the treatment options available in the literature, alignment of transposed teeth would not be recommended. This is because the reported case presented with transposition affecting canine in labial version without enough space for alignment. Despite reaching less favorable outcomes when the order of teeth is not corrected,^25^ a number of authors opt for such orthodontic therapy, which is rendered simpler. They, thus, recommend correction of pseudo or incomplete transposition only,^7^ ^,^ ^26^ ^,^ ^27^ so as to prevent root resorption, recession and hard-to-control mechanics. In the case reported herein, significant root resorption was absent at treatment completion, with only generalized rounding of root apices being found.

Orthodontic correction of transposed teeth^11,12,28,29^ was not carried out in this patient due to lack of space in the maxilla. Extraction of premolars would be necessary to correct transposition. Orthodontic correction of tooth transposition is not clinically feasible for all cases. This is because factors such as age, occlusion, patient’s compliance, tooth inclination, initial root positioning, and alveolar bone quality must be taken into account in order to move transposed teeth. Additionally, the approach demands longer treatment time as well as meticulous torque and direction of force control, so as to move the transposed teeth while preserving buccal bone cortex. Therefore, lack of space and unfavorable position of both canine and lateral incisor were decisive in opting for extraction of one transposed tooth. Of the many factors influencing the recommendation for extraction of one or both transposed teeth, the following are included: presence of trauma, little periodontal support, and deficient arch length. Since the patient presented with complete transposition, negative discrepancy in the maxilla, protrusion of maxillary incisors, and convex profile, extraction of the lateral incisor was chosen. In this context, a multidisciplinary intervention was paramount to achieve favorable aesthetics. In other words, gingivoplasty was carried out to level the gingival contour of the canines and incisors, in addition to restorative treatment performed to disguise the canine in the position of lateral incisor.

In cases of canine transposed with lateral incisor (Mx.C.I2), there are two major problems to be overcome: the ability the lateral incisor has to function as canine and the ability to disguise the canine in the position of lateral incisor. The maxillary lateral incisor is not as favorable for canine guidance, since its root is usually thin and short. Thus, group disocclusion might be recommended for non-extraction cases. Maxillary canine reshaping usually requires a combination of incisal tooth wear and composite resin or dental veneer placement.^11^ In the case reported herein, reshaping of the canine was carried out, with the latter transposed with lateral incisor both in shape and size. The procedure was performed by means of combining tooth wear and porcelain dental veneers, canine crown torque correction, so as to resemble lateral incisor torque, in addition to incorporating ideal torque for the maxillary first and second premolars mesially. Single extrusion and intrusion of the canine and first premolar, respectively, were also performed in order to achieve optimal marginal gingiva levels in the anterosuperior region, and also gingivoplasty aimed to achieve proper gingival margins. This was the treatment of choice aimed at enhancing aesthetics and function, in addition to allowing shorter treatment time.

## CONCLUSION

Tooth transposition can be successfully corrected provided that diagnosis and orthodontic planning be meticulously carried out and tooth movement be completely controlled. Meanwhile, biological limits should be respected, so as to prevent root resorption and periodontal support impairment. Tooth transposition treatment including extraction of one transposed tooth provided rather favorable outcomes, even though a multidisciplinary approach embracing Orthodontics, Periodontology and Cosmetic Dentistry was rendered necessary to achieve esthetic results, function and stability. 
